# OR-1896 increases force of contraction in the isolated human atrium

**DOI:** 10.1007/s00210-023-02592-5

**Published:** 2023-06-24

**Authors:** Lina M. Rayo-Abella, Peter Grundig, Max N. Bernhardt, Britt Hofmann, Joachim Neumann, Ulrich Gergs

**Affiliations:** 1 Institut für Pharmakologie und Toxikologie, Medizinische Fakultät, Martin-Luther-Universität Halle-Wittenberg, Magdeburger Straße 4, 06097 Halle, Germany; 2 Herzchirurgie, Medizinische Fakultät, Martin-Luther-Universität Halle-Wittenberg, Ernst Grube Straße 40, 06097 Halle, Germany

**Keywords:** OR1896, Mouse atrium, Human atrium, Phospholamban

## Abstract

OR-1896 ((R)-N-(4-(4-methyl-6-oxo-1,4,5,6-tetrahydropyridazin-3-yl)phenyl)acetamide) is the main active metabolite of levosimendan. However, nobody has reported a positive inotropic effect of OR-1896 in isolated human cardiac preparations. The mechanism of action of OR-1896 remains controversial. Hence, we wanted to know whether OR-1896 exerts a positive inotropic effect in humans and what might be the underlying mechanism. Therefore, we measured the contractile effects of OR-1896 (0.01–10 µM cumulatively applied) in isolated electrically stimulated (1 Hz) human right atrial preparations (HAP) obtained during cardiac surgery. OR-1896, given alone, exerted time- and concentration-dependent positive inotropic effects; 1-µM OR-1896 increased force by 72 ± 14.7% (*p* < 0.05, *n* = 6) and shortened the time of relaxation by 10.6 ± 3.6% (*p* < 0.05, *n* = 11) in HAP started at 0.1 µM, plateaued at 1-µM OR-1896, and was antagonized by 1-µM propranolol. The maximum positive inotropic effect of OR-1896 in human right atrial preparations was less than that of 10-µM isoprenaline. EMD 57033 (10 µM), a calcium sensitizer, enhanced the force of contraction further in the additional presence of 1-µM OR-1896 by 109 ± 19% (*p* < 0.05, *n* = 4). Cilostamide (10 µM), an inhibitor of phosphodiesterase III given before OR-1896 (1 µM), blocked the positive inotropic effect of OR-1896 in HAP. Our data suggest that OR-1896 is, indeed, a positive inotropic agent in the human heart. OR-1896 acts as a PDE III inhibitor. OR-1896 is unlikely to act as a calcium sensitizer in the human heart.

## Introduction

**Fig. 1:**
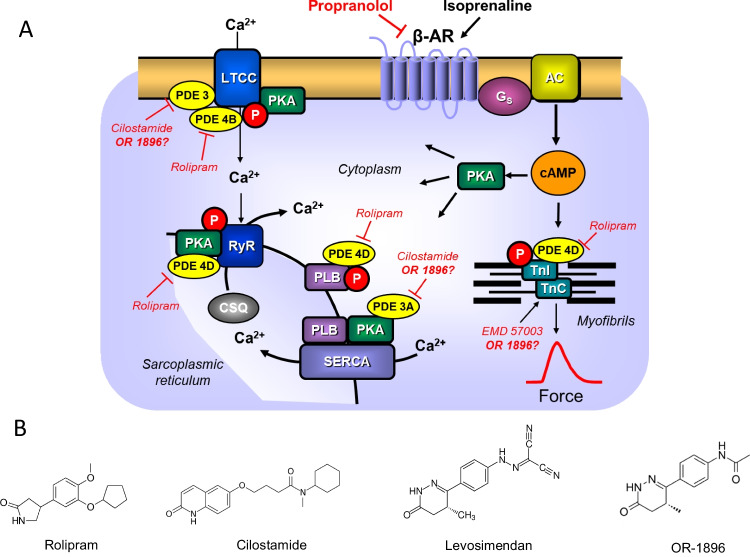
Scheme: Potential mechanism(s) of action of OR-1896 in the human and mouse cardiomyocytes. Stimulation of the activity of β-adrenoceptors (β-AR) by endogenous noradrenaline or exogenous isoprenaline leads via stimulatory GTP-binding proteins (Gs) to an increase of adenylyl cyclase (AC) activity. Adenylyl cyclase increases the formation of 3´,5´-cyclic-adenosine-mono-phosphate (cAMP) that stimulates cAMP-protein kinase (PKA). PKA phosphorylates and thus activates phospholamban (PLB) at the amino acid serine 16, the inhibitory subunit of troponin (TnI), the ryanodine receptor (RYR), and the L-type calcium channel (LTCC). The formed cAMP can be degraded to inactive 5´-AMP and pyrophosphate by isoenzymes of the phosphodiesterase family of proteins (PDE). Cilostamide and rolipram (bottom: structural formulae) inactivate phosphodiesterase 3 and 4, respectively. The β-adrenoceptor is blocked by β-adrenoceptor blockers like timolol or propranolol. Calcium cations (Ca^2+^) are stored on calsequestrin (CSQ) in the sarcoplasmic reticulum and are released via RYR from the sarcoplasmic reticulum (SR). These released calcium cations bind to troponin C on thin myofilaments, and as a result, systolic force is augmented. Typical calcium sensitizers like EMD 57033 act by generating at a given level of free Ca2 + more force of contraction. In cardiac diastole, calcium cation concentrations fall because calcium cations are pumped into the SR via the SR-calcium ATPase (SERCA). The activity of SERCA is increased when phospholamban is phosphorylated on amino acid serine 16. OR-1896 (bottom: structural formula) might act by increasing the sensitivity for calcium cations of troponin C in the thin myofilaments or might inhibit phosphodiesterases in the heart

In the heart, positive inotropic effects can be achieved with phosphodiesterase inhibitors (PDE, scheme in Fig. 1A). PDE III inhibitors like milrinone exerted positive inotropic, lusitropic, and positive chronotropic effects (reviews: Scholz and Meyer 1986; Schmitz et al. 1989, 1992). However, more heart failure patients died in the milrinone group than in the placebo group (Packer et al. 1991).

Hence, new mechanisms of inotropic action, independent of an elevation of Ca^2+^ levels in the cytosol of cardiomyocytes (Fig. 1A), have thereafter been sought. For instance, so-called calcium sensitizers (Rüegg et al. 1984, Ventura et al. 1992), like levosimendan, OR-1896 (Haikala et al. 1995, 1997, structural formula in Fig. 1B), CGP 48506 (Neumann et al. 1996, Zimmermann et al. 1996), or EMD 57033 (Neumann et al. 1995; Uhlmann et al. 1995), were studied. These calcium sensitizers raise the affinity of myofilaments for calcium cations, but some of these drugs retained PDE-inhibitory activity.

Levosimendan was marketed as a pure calcium sensitizer (Haikala et al. 1995). In contrast to this view, in guinea pig cardiac preparations, (i) levosimendan accelerated cardiac relaxation in the guinea pig heart (Boknik et al. 1997), (ii) levosimendan exerted a positive chronotropic effect in spontaneously beating right atrial preparations from guinea pig hearts (Boknik et al. 1997), and (iii) levosimendan elevated cAMP concentrations, increased the phosphorylation state of phospholamban, the inhibitory subunit of troponin and C-protein, and enhanced the current through the L-type calcium channel in cardiomyocytes from guinea pig ventricles (Boknik et al. 1997, Virag et al. 1996). In contrast to levosimendan, a pure calcium sensitizer, namely CGP 48506, prolonged cardiac relaxation time and did not increase cAMP content nor phospholamban phosphorylation in guinea pig cardiac preparations (Zimmermann et al. 1996, 1998).

Consistent with the findings in guinea pig cardiac preparations, the phosphodiesterase III inhibitor cilostamide (Fig. 1B) blocked any positive inotropic effect of levosimendan in ventricular muscle strips from failing human hearts (Ørstavik et al. 2014). Thus, the positive inotropic effect of levosimendan seems to result from the inhibition of phosphodiesterase III in the failing human ventricle (Ørstavik et al. 2014). Their conclusion was supported by their finding that the β-adrenoceptor antagonist timolol reversed the positive inotropic effect of levosimendan in human ventricular preparations (Ørstavik et al. 2014). More recently, we could confirm these data in human atrial preparations (Rayo Abella et al. 2022a, b): Levosimendan increased force of contraction in human atrial preparations; this increase was accompanied by an elevation in the phosphorylation state of phospholamban, and both effects were abrogated by propranolol. Moreover, cilostamide pretreatment hindered levosimendan to increase the force of contraction in isolated human atrial preparations (Rayo Abella et al. 2022a, b).

OR-1896, the main metabolite of levosimendan, when given alone, increased the force of contraction in rat papillary muscles (Ørstavik et al. 2015). One noted that OR-1896 induced a positive inotropic effect starting at 0.1-µM OR-1896 that plateaued at 1 µM (Ørstavik et al. 2015). This effect, the positive inotropic effect of OR-1896, was augmented by rolipram (10 µM, a phosphodiesterase IV inhibitor) but blocked by the PDE III inhibitors milrinone (1 µM) or cilostamide (1 µM, Ørstavik et al. 2015). The positive inotropic effect of OR-1896 could not be augmented by additionally applied EMD57033 (3 µM, a calcium sensitizer). OR-1896 was less effective than isoprenaline (100 µM, Ørstavik et al. 2015). OR-1896, alone, exerted a lusitropic effect that was potentiated by rolipram (Ørstavik et al. 2015). In the presence of a β-adrenoceptor antagonist, OR failed to increase the force of contraction in rat papillary muscles (Ørstavik et al. 2015). OR-1896 did not alter the potency of Ca^2+^ to raise the force of contraction of rat papillary muscles (Ørstavik et al. 2015). OR-1896 inhibited PDE activity in the rat heart with a similar efficacy as cilostamide (Ørstavik et al. 2015). Consistent with a PDE inhibition, the authors noted that cAMP was increased after the addition of OR-1896 to rat ventricular cardiomyocytes (Ørstavik et al. 2015). The authors concluded that in rat papillary muscles, OR-1896 increased the force of contraction by inhibition of PDE III (Ørstavik et al. 2015). However, they did not test human tissue.

In patients, OR-1896 has a longer half-life than levosimendan, which amounts to about 1 h; in contrast, the half-life of OR-1896 is about 70–80 h (Koskinen et al. 2008, Grześk et al. 2022). Plasma levels of OR-1896 are higher in rapid acetylators of OR-1896 than in slow acetylators of OR-1896 (Antila et al. 2004). Hence, in some patients, OR-1896 might be clinically especially relevant in their response to levosimendan treatment. There are data from skinned fibers that OR-1896 is a calcium sensitizer also in a human ventricle (Papp et al. 2004). Usually, OR-1896, on a molar basis, is less potent than levosimendan. For instance, levosimendan was more potent to increase the rate of force development in living rats (Segreti et al. 2008). Likewise, OR-1896 was less potent than levosimendan to inhibit PDE III (levosimendan: 2.5 nM, OR-1896: 94 nM) and PDE IV (levosimendan: 25 µM, OR-1896: 286 µM) in the guinea pig heart. However, levosimendan and OR-1896 were about equipotent (levosimendan 15 nM, OR-1896: 25 nM) to raise intraventricular developed pressure in Langendorff perfused guinea pig hearts (Szilágyi et al. 2004). Both levosimendan and OR-1896 raised the rate of pressure development by about 25% and were thus equieffective (Szilagyi et al. 2004). OR-1896 is assumed to contribute to the clinical effect of levosimendan in heart failure patients. OR-1896 also has vasodilatory properties that are explained in part by the opening of the potassium channel and a cAMP increase in the vasculature (review: Burkhoff et al. 2021). These vasodilatory and ancillatory effects are thought to be beneficial in patients with heart failure.

The group of Masao Endoh generated data that OR-1896 in dog papillary muscles OR-1896 increased the force of contraction at least in part by elevating cytosolic-free calcium ions (Takahashi et al. 2000). Moreover, the positive inotropic effect of OR-1896 was antagonized in dog papillary muscles by carbachol, suggesting to the authors that also a cAMP-dependent component contributes to the positive inotropic effect of OR-1896 in the dog heart (Takahashi et al. 2000).

We find it important to understand better the mechanism of action of the active metabolite of levosimendan, namely OR-1896, in order to better understand how the long-term actions of levosimendan on the human heart come about mechanistically. However, prior to the present study, the effects of OR-1896 in isolated human atrium or human ventricular preparations on the force of contraction were unknown. For comparison, we performed similar experiments on cardiac atrial preparations from mice. These mouse data have the potential benefit to study the effect of OR-1896 on sinus node function in isolated preparations which are not readily feasible in human hearts. Thence, we studied the hypothesis that OR-1896 increased contractile function in isolated electrically paced human right atrial muscle strips. Furthermore, we asked whether this effect is altered by the concomitant application of a PDE III inhibitor and is accompanied by increased phospholamban phosphorylation.

## Methods

### Contractile studies in mice

In brief, wild-type mice were sacrificed, the thorax was opened, and the heart was mobilized and cut from the ascending aorta to make sure the right atrium was not damaged. Then, the whole heart was transferred to a dissection chamber filled with gassed Tyrode’s solution at room temperature. Right or left atrial preparations were isolated and mounted in organ baths, as described by Gergs et al. (2013, 2017, 2019a, b) and Neumann et al. (2003). Force was detected under isometric conditions, amplified and fed into a digitizer, and quantified by commercial software (LabChart 8, AD Instruments, Spechbach, Germany).

### Contraction studies in the human atrium

These experiments were performed as reported repeatedly (e.g., Gergs et al. 2009; Neumann et al. 2021a, b). In brief, during cardiac surgery, at the site where the cannula for extracorporeal circulation entered the heart, small muscle strips were obtained from the right atrium. Patients were aged between 48 and 72 years. Medication included acetylsalicylic acid, nitrates, diuretics, β-adrenoceptor blockers, and anticoagulants. Atrial trabeculae were dissected and mounted in an organ bath and electrically stimulated (1 Hz) and processed like mouse preparations (see above).

### Western blotting

The process of sample homogenization, protein concentration measurement, electrophoresis, antibodies incubation, and signal quantification were performed following our previously published protocols with slight modifications (Boknik et al. 2018; Gergs et al. 2009; Gergs et al. 2019a). Electrophoresis was performed in Novex™ 4–20% “Tris–Glycine Plus Midi Protein Gels” (Invitrogen, Thermo Fisher Scientific, Waltham, Massachusetts, USA). The run was performed at 4 °C for approximately 1 h at 120 V in the “NuPAGE MES SDS Running Buffer” (Thermo Fisher Scientific, Waltham, Massachusetts, USA) using an XCell4 SureLock™ Midi-Cell chamber (Life Technologies by Thermo Fisher Scientific, Waltham, Massachusetts, USA). Protein transfer into membranes (Amersham™ Protran, GE Healthcare, Chicago, Illinois, USA) was performed at 2 A for 2 h at 4 °C. Membrane blocking for 1 h at room temperature was followed by overnight incubation at 4 °C with the primary antibody for serine 16-phosphorylated phospholamban (1:5000; catalog number: A010-12AP; PLB Ser16; Badrilla, Leeds, UK), SERCA2 ATPase (1:20.000; catalog number: ab2861; abcam, Cambridge, UK), and phospho-troponin I (1:5000; catalog number: 4004; Ser23/24; cell signaling technology, Leiden, the Netherlands). While calsequestrin antibody was used as a loading control (1:20.000; product number: ab3516; abcam, Cambridge, UK). Visualization of the signals was performed by using a chemiluminescent HRP substrate (Immobilon™ Western, Millipore, Merck; Darmstadt, Germany) and a digital imaging system (Amersham ImageQuant 800; Cytiva Europe GmbH, Freiburg im Breisgau, Germany).

### Data analysis

Data were treated as in most of our previous studies (e.g., Gergs et al. 2019, Neumann et al. 2021a, b). Shown are the means ± standard error of the mean. Statistical significance was estimated using the analysis of variance (ANOVA), followed by Bonferroni’s *t*-test or Student’s *t*-test as appropriate. A *P*-value of less than 0.05 was considered significant. Experimental data for agonist-induced positive inotropic and chronotropic effects were analyzed by fitting sigmoidal curves to the experimental data with GraphPad Prism 5.0. All other statistical analyses were performed as indicated in the figures and tables.

### Drugs and materials

(-)-Isoprenaline ( +)-bitartrate, rolipram, propranolol, and cilostamide were purchased from Sigma-Aldrich (Deisenhofen, Germany). OR-1896 ((R)-N-(4-(4-methyl-6-oxo-1,4,5,6-tetrahydropyridazin-3-yl)phenyl)acetamide) was from Biozol (Munich, Germany). All other chemicals were of the highest purity grade commercially available. Deionized water was used throughout the experiments. Stock solutions were freshly prepared daily.

## Results

### Mouse

#### Force of contraction in left atrial preparations

**Fig. 2:**
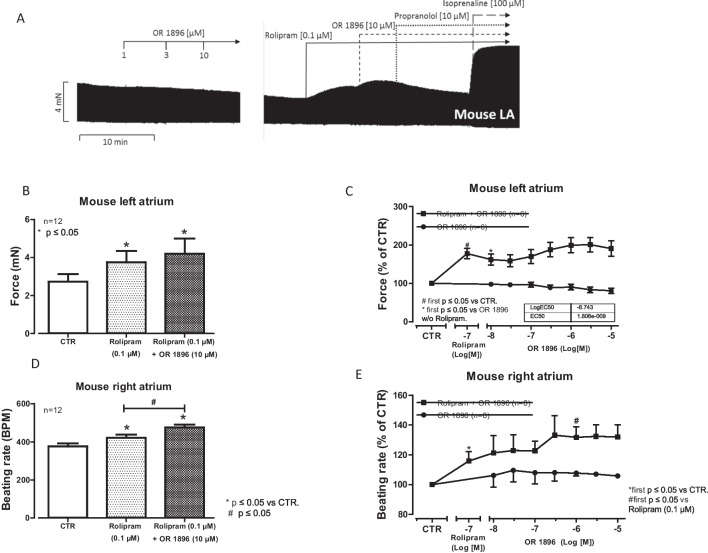
Positive inotropic and chronotropic effects of OR-1896 in the mouse left and right atrium, respectively, only in the presence of rolipram. (**A**) Original recordings: OR-1896 ((-)-(R)-[[4-(1,4,5,6-tetrahydro-4-methyl-6-oxo-3-pyridazinyl)phenyl]hydrazono]propane-dinitrile) (1 µM to 10 µM) was cumulative applied alone or the presence 0.1-µM rolipram ((RS)-4-(3-cyclopentyloxy-4-methoxy-phenyl)pyrrolidin-2-on). Rolipram was used to inhibit the activity of phosphodiesterase IV. The positive inotropic effect of OR-1896 in the presence of rolipram was abrogated by the subsequent application of 10-µM propranolol, and the maximal effect on inotropy was reached after the application of 100-µM isoprenaline. Vertical bars indicate force in milli Newton (mN). Horizontal bars indicate time in minutes (min). (**B**) Bar graph showing the force of contraction by not cumulatively applied Rolipram (0.1 µM) alone and by OR-1896 (10 µM) in the presence of Rolipram (0.1 µM) in electrically stimulated left atrial preparations from mouse hearts. The positive inotropic effects of rolipram and rolipram in an additional supply of OR-1896 are significant (*p* < 0.05, Student’s *t*-test) in relation to the point prior to the administration of any substance (CTR). The number of individual experiments is represented by the value of *n*. Ordinates indicate the effect of rolipram and OR-1896 in the presence of rolipram on the force of contraction in milli Newton (mN). (**C**) Summarized concentration–response curves for increasing concentrations of OR-1896 on the force of contraction alone (circles) after the addition of 0.1-µM rolipram (squares) to the organ bath in isolated electrically stimulated left atrial preparations from mouse hearts. The ordinates indicate the effects of OR-1896 and rolipram on contraction force as a percentage of the pre-drug value (% of CTR). Abscissae: negative decadic logarithm of the concentrations of rolipram and OR-1896. # indicates first concentration with *p* < 0.05 versus CTR, and * indicates first concentration with *p* < 0.05 versus OR-1896 without rolipram (Student’s t-test). The number in brackets indicates the number of experiments. (**D**) Bar chart indicating the increase in the beating rate (BPM: beats per minute) in isolated spontaneously beating right atrial preparations from mouse hearts. This increase can be seen by the symbol * (*p* < 0.05, Student’s *t*-test) compared to control conditions (pre-drug point: CTR) for rolipram (0.1 µM) and the combination of rolipram (0.1 µM) and OR-1896 (10 µM). Similarly, there is a difference in the beating rate between rolipram (0.1 µM) alone and rolipram with a late addition of 10-µM OR-1896 to the organ bath (#*p* < 0.05, Student’s *t*-test). The number of experiments is represented by the value of *n*. (**D**) Summary data of the positive chronotropic effect of a cumulative dose of OR-1896 (0.01 µM–10 µM) only in the presence of 0.1-µM rolipram in isolated right atrial preparation of spontaneously beating mouse heart. The ordinates indicate the effect of OR-1896 with or without rolipram as a percentage of the pre-drug value (% of CTR). Abscissae: negative decadic logarithm of rolipram and OR-1896 concentrations. *p < 0.05 versus CTR (Student’s *t*-test). The number in brackets indicates the number of individual experiments

OR-1896 cumulatively applied, when given alone, from 10 nM to 10 µM (the highest concentration tested in this study), did not raise contractility in electrically driven (1 Hz) left atrial preparations of mouse hearts compared to control conditions (OR-1896 alone: original recording; Fig. 2A). However, when first 0.1-µM rolipram (a phosphodiesterase IV inhibitor, Fig. 1A,B) was applied, rolipram itself an increased force of contraction to some extent (Fig. 2A) and additionally applied OR-1896 augmented force of contraction further (Fig. 2A), in left atrial (LA) preparations from mice. The positive inotropic effects of OR-1896 in the presence of rolipram were diminished by 10-µM propranolol (Fig. 2A). The effects of OR-1896 were time-dependent and concentration (10 nM to 10 µM)-dependent (Fig. 2A). The positive inotropic effect reached its maximum at 1-µM OR-1896 (Fig. 2A). However, subsequently applying 10-µM isoprenaline increased force of contraction in LA, further indicating that OR-1896 under our experimental conditions was less effective than isoprenaline to raise a force of contraction (Fig. 2A). In several initial experiments, we gave rolipram and then non-cumulatively only one concentration of OR-1896 (Fig. 2B). An effect of rolipram was apparent, but OR-1896 did not increase force further. We decided to construct concentration–response curves for OR-1896, hoping to detect at least a small positive inotropic effect. But as seen in Fig. 2C, the effects were too small to gain significance even under these more favorite conditions. Several such experiments are summarized in Fig. 2B.

#### Beating rate in right atrial preparations

One could argue that our concentrations of OR-1896 or rolipram were too low to detect functional alterations in the mouse atrium. However, this does not seem to be an acceptable generalization: Indeed, OR-1896 alone does not affect spontaneously beating right atrial preparations (Fig. 2E). A concentration of 0.1-µM rolipram by itself exercised a positive chronotropic effect of its own (Fig. 2E). When one applied OR-1896 (non-cumulatively: Fig. 2D; cumulatively: Fig. 2E) in addition or rolipram, OR-1896 increased the beating rate in spontaneously beating mouse right atrial preparations.

### Human studies

#### Force of contraction in isolated human atrial preparations

**Fig. 3:**
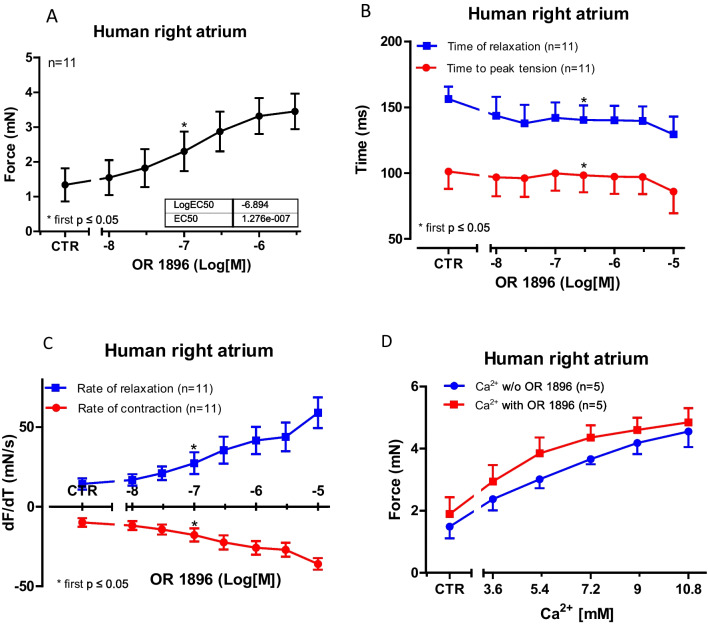
Inotropic effects of OR-1896 in human atrium. Concentration-dependent effects of increasing concentrations of OR-1896 (0.01 µM–10 µM) on the force of contraction, times of contraction or rate of tension, in electrically stimulated muscle strips from the human right atrium are summarized in **A**, **B**, or **C**. OR-1896 was cumulatively applied. Ordinates indicate the effect of OR-1896 on the force of contraction in milli Newton (mN: A), on time to peak tension in milliseconds (ms: E), on time of relaxation (ms: E), on rate of tension development (in mN/s: C), and on rate tension of relaxation (mN/s: C). Abscissae: negative decadic logarithm of the concentrations of OR-1896. *p < 0.05 versus control conditions (pre-drug point: CTR; Student’s *t*-test). The number in brackets indicates the number of individual experiments. (**D**) The positive inotropic effects of increasing Ca^2+^ concentrations were not potentiated by 1-µM OR-1896. Ordinates indicate the effect of Ca^2+^ on the force of contraction in milli Newton (mN), and the abscissae show the molar concentrations of Ca^2+^. The number in brackets indicates the number of experiments

In contrast to the findings in mouse atrial preparations (Fig. 2C), cumulatively applied OR-1896 alone exerted a concentration-dependent positive inotropic effect in human atrial preparations (Fig. 3A). In these human atrial preparations, OR-1896 concentration-dependently reduced time to peak tension (Fig. 3B) and time of relaxation (Fig. 3B). Moreover, OR-1896 concentration-dependently increased the rate of tension development and the rate of tension relaxation (Fig. 3C). In addition, we detected no significant calcium sensitizing properties: When we raised Ca^2+^ concentrations in the organ bath, we elevated thereby force of contraction. However, these effects of Ca^2+^ were not potentiated by OR-1896 (Fig. 3D).

**Fig. 4:**
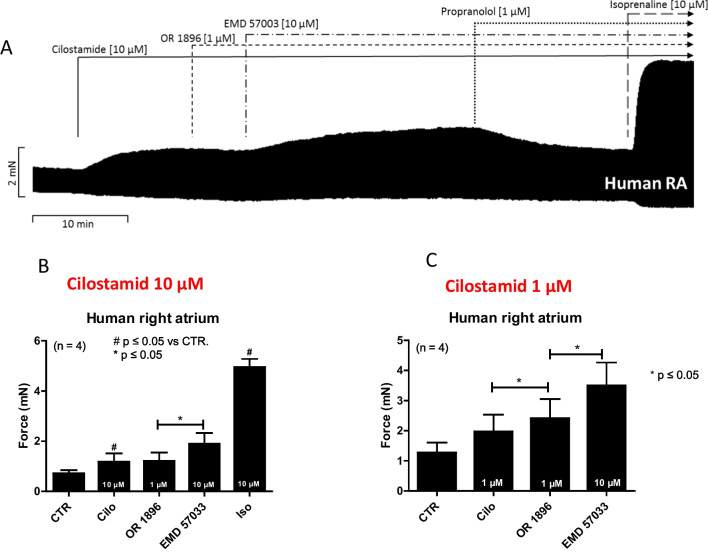
The positive inotropic effect of OR-1896 in the human atrium does not necessitate the concomitant inhibition of phosphodiesterase IV and is abrogated in the concomitant inhibition of phosphodiesterase III. (**A**) Original recordings: in electrically stimulated muscle strips from the human right atrium. Effects of 1-µM OR-1896 in the additional presence of 10-µM cilostamide. The effect was increased by 10 µM of EMD 57003 and abrogated by 1-µM propranolol. Subsequent application of a very high concentration of isoprenaline (10 µM) surmounted the effect of propranolol, indicating that the sample was properly contracting and that the efficacy of 10-µM isoprenaline was superior to that of 1-µM OR-1896. Vertical bars indicate force in milli Newton (mN). Horizontal bars indicate time in minutes (min). Samples were incubated with 10 µM (**B**) or 1 µM (**C**) cilostamide (Cilo) and in the additional presence of 1-µM OR-1896, EMD 57003 (10 µM) and isoprenaline (10 µM). Combining several such experiments, the mean values and SEM are seen in Fig. 5B,C, respectively. While 1-µM cilostamide (**C**) increased the force of contraction in human atrial preparations to some extent and OR-1896 exerted a small addition positive inotropic effect, 10-µM cilostamide (**B**) impaired any positive inotropic effect of subsequently added 1-µM OR-1896. Vertical bars indicate force in milli Newton (mN). #*p* < 0.05 versus control conditions (pre-drug point: CTR) and *p < 0.05 (ANOVA and Bonferroni’s multiple comparison test). The number in brackets indicates the number of individual experiments

In contrast, while 1-µM cilostamide increased the force of contraction in human atrial preparations to some extent and OR-1896 exerted a small additional positive inotropic effect (Fig. 4C), 10-µM cilostamide impaired any positive inotropic effect of subsequently added 1-µM OR-1896 (Fig. 4B). This is in line with findings by others in rat ventricular strips (Ørstavik et al. 2015). The positive inotropic effects of OR-1896 (1 µM) were abrogated by 1-µM propranolol in human atrial preparations consistent with the action OR-1896 as a PDE III inhibitor in human atrial tissue (Fig. 1A). After maximal stimulation by 1-µM OR-1896, the force of contraction was augmented by additionally applying EMD 57033 (Fig. 4B,C), a calcium sensitizer, suggesting that in the human atrium, EMD 57033 and OR-1896 have different mechanisms of action (Fig. 1A). These data combined argue against a calcium sensitizing effect of OR-1896, at least under our experimental conditions in human atrium. Moreover, the maximum inotropic effect of OR-1896 was lower than that of 10-µM isoprenaline (Fig. 4A), in line with reports for levosimendan in human ventricular preparations (Ørstavik et al. 2014) and in human atrial preparation (Rayo Abella et al. 2022a, b).

#### Phospholamban phosphorylation in the human atrium

**Fig. 5:**
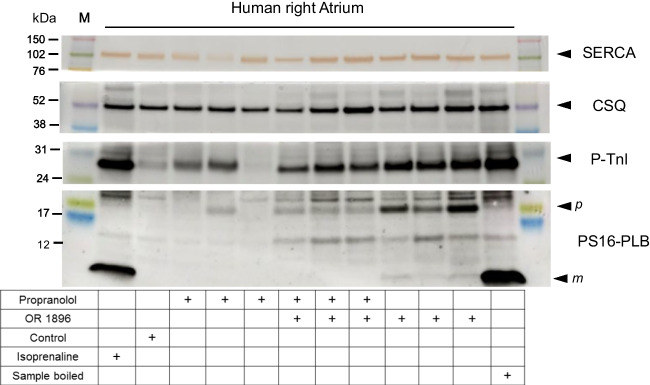
OR-1896 alone increases the phosphorylation of phospholamban in the human heart. Western blot of phosphorylated phospholamban at serine 16 and the phosphorylated inhibitory subunit of troponin (TnI) in contracting human atrium. Horizontal arrows indicate the apparent molecular weight of phospholamban phosphorylated at serine 16 (PS16-PLB) or phosphorylated inhibitory subunit of troponin (P-TnI), calsequestrin (CSQ), and SERCA were used as a loading control. OR-1896 1 µM or additional 1-µM propranolol was added to contracting human right atrial muscle strips, as indicated and freeze-clamped at the plateau of the positive inotropic effect. Human atrial samples were homogenized, subjected to gel electrophoresis, and transferred to nitrocellulose membranes. Membranes were cut horizontally as indicated and were incubated with primary and secondary antibodies (see Methods), and signals were scanned, and a typical blot is presented here. Apparent molecular weight standards are depicted in the left-hand first lane. Samples treated only with propranolol (10 µM) were included in the blot for time control purposes in the experiment. As a positive control, samples treated with 10-µM isoprenaline are shown at the ends, and as a negative control, an untreated sample (Control) was included in the blot. Boiled means that the isoprenaline-treated sample was brought to the temperature of 95 °C for 10 min. Please note the mobility shift in the boiled sample, confirming that we really detected phospholamban

1-µM OR1896 alone (that is, non-cumulatively applied) increased the phosphorylation state of phospholamban at serine 16 in contracting human atrial strips that were freeze-clamped at the maximum of the positive inotropic effect (Fig. 5). The positive inotropic effects of OR1896 in human atrial strips were completely reversed by 1-µM propranolol (Fig. 4A). In the same atrial preparations where force was recorded and that were freeze-clamped, 1-µM propranolol reduced the increase in the phosphorylation state of phospholamban induced by 1-µM OR1896 (Fig. 5), arguing against the action of OR-1896 as a pure calcium sensitizer in the human atrium.

## Discussion

The first new finding of the present paper is that OR-1896 has a positive inotropic effect in the human heart, in this case, the isolated human atrium. This is important because OR-1896 is the main active metabolite of levosimendan. Levosimendan is currently recommended in guidelines for some patients with heart failure (Papp et al. 2020). A second new finding is that the mechanism in the human heart of OR-1896 can be attributed mainly to the inhibition of phosphodiesterase III. This is important clinically because this kind of mechanism may explain any arrhythmias seen in patients treated with levosimendan (Papp et al. 2020).

### Contractile data in mouse left atrium

The present data on mouse left atrium are somewhat divergent from those in rat ventricular preparations by others (Ørstaviket al. 2015). These authors, unlike ourselves, noted that OR-1896, when given alone, was effective to raise the force of contraction in rat ventricular muscle strips (Ørstavik et al. 2015). We would argue that our observations are in line with the interpretation that OR-1896 heightens the force of contraction in the mouse heart through inhibition of phosphodiesterase III, keeping in mind that PDE III is relatively unimportant for force regulation in the mouse heart. While the human heart mainly expresses (detected as protein in Western blots and in PDE activity assays) PDE III, the mouse heart expresses mainly PDE IV (Abi-Gerges et al. 2009). In accordance with these biochemical data, typical PDE III inhibitors like cilostamide are very potent and effective in raising the force in human cardiac muscle preparations, whereas typical PDE IV inhibitors like rolipram alone do not raise the force of contraction in human cardiac preparations (discussed in Neumann et al. 2021a). In the mouse, we find the opposite pattern. Cilostamide does not increase the force of contraction in mouse atrial preparations, whereas rolipram is very potent and efficient in raising the force of contraction in the mouse atrium (Neumann et al. 2019, 2021a). OR-1896 alone in rat ventricular cardiomyocytes has been shown to increase cAMP levels (Ørstavik et al. 2015); thus, it is plausible that they observed a small positive inotropic effect with OR-1896 alone. Interestingly (in isolated rat ventricular cardiomyocytes), after pre-incubation with cilostamide, OR-1896 did not increase cAMP levels any further, supporting the assumption that OR-1896 increased cAMP levels already via PDE III inhibition in rat ventricle (Ørstavik et al. 2015). Moreover, the authors failed to detect Ca^2+^ sensitizing properties of OR 1896 in rat ventricles, but noted that OR-1896 could inhibit the activity of PDE III in rat ventricular preparations (Ørstavik et al. 2015).

### Chronotropic effects of OR-1896 in mouse right atrium

The present work clearly shows that OR-1896 increased the beating rate in the mouse’s right atrium. These positive chronotropic effects in mouse strongly argue against an inotropic action of OR-1896 as a calcium sensitizer. This is so because, typically, calcium sensitizers neither increase nor decrease the beating rate (e.g., Zimmermann et al. 1996), presumably because they do not increase cAMP in any compartment of the heart, because they do not stimulate cAMP production and do not inhibit cAMP degradation. Moreover, the effect in the sinus node also seemed to result from the amplification of cAMP levels. Endogenously produced or at least present noradrenaline may activate the β-adrenoceptor in the mouse sinus node to a certain extent, and this effect was amplified by the PDE-inhibitory properties of OR-1896. Indeed, our findings on the beating rate in mouse right atrial preparations are consistent with the view that OR-1896 acts via elevation of cAMP also in sinus node cells. This observation concurs with the hypothesis that also, in the mouse sinus node, OR-1896 operates as a phosphodiesterase III inhibitor: Fittingly, pre-incubation with rolipram amplified the positive chronotropic effect of OR-1896 because now PDE III and PDE IV are inhibited and only then a functional effect on the mouse sinus node can occur.

### Effects of OR-1896 on the human atrium

In contrast to mouse atrial preparations, in isolated electrically stimulated human atrial preparations, OR-1896 alone elevated the force of contraction and shortened, for instance, relaxation time. We assume this is probably due to a surge in the phosphorylation state of phospholamban that we reported in Fig. 5. Cardiac relaxation is thought to be enhanced by phosphorylation of phospholamban (Hamstra et al. 2020) and phosphorylation of the inhibitory subunit of troponin (Vetter et al. 2018). Un-phosphorylated phospholamban inhibits the activity of SERCA (Ca^2+^-pump of the sarcoplasmic reticulum (SR): Fig. 1). When cAMP is increased, cAMP-dependent protein kinase phosphorylates phospholamban at the amino acid serine 16 (Hamstra et al. 2020). From mutational studies in vitro and in knock-in mice, we know that this phosphorylation disinhibits SERCA activity (Hamstra et al. 2020). This increased activity of SERCA has at least two consequences: On the one hand, SERCA pumps calcium ions faster out of the cytosol, and thus, the calcium ions leave the vicinity of myofilaments and myofilaments relax faster. On the other hand, because more calcium ions were pumped by SERCA into the SR, more calcium ions are now stored in the SR. When the next heartbeat occurs, more calcium ions are present in the SR, and therefore, more calcium ions are released (Hamstra et al. 2020). This contributes to the subsequent rise in the force of contraction and leads to a positive inotropic effect. Phosphorylation of TnI can reduce the affinity of myofilament to cytosolic calcium ions: This can contribute to faster heart muscle relaxation (Vetter et al. 2018).

Pure calcium sensitizers do not increase the phosphorylation state of phospholamban at serine 16 (Zimmermann et al. 1996). Likewise, pure calcium sensitizers do not increase the rate of relaxation, and they do not shorten the time of relaxation: In contrast, pure calcium sensitizers even prolong the duration of contraction in the guinea pig ventricle (Zimmermann et al. 1996) but also in the human ventricle (Neumann et al. 1996).

Positive inotropic effects of OR-1896 in human atrial preparations have, to the best of our knowledge, not been reported before. On the other hand, our interpretation is supported by the fact that OR-1896 shortened the time of relaxation in rat ventricular muscle strips (Ørstavik et al. 2015).

Finally, our observations that propranolol attenuated the positive inotropic effect of OR-1896 and reduced the OR-1896-induced increase in phospholamban phosphorylation in human atrial preparations also contradict the view that OR-1896 is a calcium sensitizer in the human heart.

## Conclusion

The present data strongly suggest that the positive inotropic and relaxant effects of OR-1896 in the isolated human right atrium result from the lowered activity of phosphodiesterase III.

## Data Availability

The data of this study are available from the corresponding author upon reasonable request.
